# Relative snowpack response to elevation, temperature and precipitation in the Crown of the Continent region of North America 1980-2013

**DOI:** 10.1371/journal.pone.0248736

**Published:** 2021-04-13

**Authors:** Len Broberg

**Affiliations:** Environmental Studies Program, University of Montana, Missoula, Montana, United States of America; Universitat Zurich Institut fur Volkswirtschaftslehre, SWITZERLAND

## Abstract

Water availability in western Canada and the United States is dependent on the accumulation of snowpack in the montane regions and threatened by increased winter temperature and more precipitation as rain linked to climate change. In order to make reasoned decisions to adapt to climate change managers require knowledge of the role of temperature and precipitation in SWE development and data to distinguish the relative retention response of snowpack regions to expected temperature and precipitation regime shifts at the watershed scale. Using the Daymet interpolated 1 km^2^ dataset, effects of elevation, temperature (T_max_, T_min_ and T_avg_) and precipitation on April 1 SWE in the Crown of the Continent were tested by linear regression and Kendall correlation. Changes in Daymet estimated snow water equivalent (SWE) in response to increased temperatures and changes in precipitation were estimated in two ways: 1) comparing April 1SWE in the 11 warmest (mean T_max_ February) and driest (mean precipitation January to March) years with the 22 cooler/wetter years 1981–2013 and 2) SWE retention from April 1 to June 1 over the period 1980 to 2013 across 120 watersheds in a major continental headwater region, the Crown of the Continent of North America. Historical analysis of period warm year April 1 SWE was assumed to indicate the recent impact of warmer winter temperatures. Changes in snowpack April 1 to June 1 reflected likely effects on peak runoff and were, therefore, also relevant for future climate change adaptation considerations. Winter (JFM) precipitation proved more influential than temperature in shaping April 1 SWE response at the regional scale. Of the three factors, elevation was most positively associated with April 1 SWE at the watershed scale. Temperature and precipitation influenced SWE accumulation and persistence at the watershed scale, but higher precipitation was more closely associated with higher April 1 SWE retention. Ranking of watershed snowpack retention in warm and dry years, combined with spring snowpack retention offers data to assist identification of watersheds with greatest snowpack persistence in the face of anticipated climate change effects.

## Introduction

Water resources are critical to the sustenance of natural and human communities worldwide. Mountainous regions function as water towers that feed lower elevation ecosystems, agriculture and domestic use [1]. In western North America water availability depends on winter/spring snowpack accumulation in mountainous areas and subsequent snowmelt [2–5]. The accumulation of winter snowpack and spring ablation influences the timing of spring pulse streamflow [6], seasonal streamflow volumes [5, 7], and the sensitivity of streams to summer air temperature extremes [8, 9]. Declines in associated hydrologic processes, therefore, can have detrimental impacts on biological communities [10] and water resource availability for human uses [5, 9]. Given the dependence of multiple levels of ecological and human systems on snowpack, understanding how temperature/precipitation variation might affect snowpack in the future is paramount.

The critical measures of snowpack extent, depth and water content are driven by the two major processes of accumulation and ablation. In turn, each of these processes are influenced by multiple factors. Snowpack accumulation results from global and continental scale climate processes [11] realized at regional and local scales. Regional precipitation and temperature regimes [2, 12] coupled with landscape and landform factors such as elevation [13], slope [14], aspect [14], canopy [15] and latitude [11], ultimately drive local scale variation of snowpack accumulation. Heterogeneity of snowfall accumulation in complex topography is prevalent due to regional and local storm tracks [16, 17], orographic precipitation effects, vegetation structure [18, 19] and the effects of wind distribution of snow [16] at various scales. Snowpack ablation includes loss of snowpack volume and coverage through the transition of water from solid to liquid state (melt) linked to temperature increase above freezing and sublimation. Fine-scale topographic heterogeneity can affect rates of melt and sublimation [20, 21]. Snow accumulation and ablation in the Rocky Mountains spanning the US-Canada border also responds to the Pacific Decadal Oscillation (PDO), which is also linked to the El Niño Southern Oscillation (ENSO) patterns of Pacific climate variation occurring over decadal and annual scales respectively [22–25].

Both the scientific and management community often use snow water equivalent (SWE), the amount of water in the snow, as a measure of accumulation [24]. Snow water equivalent is unaffected by short term variation in snow depth from settlement and compaction and reflects the resource value most critical to water availability. Loss of snowpack has been characterized by measures such as snow cover depletion (SCD) [25] or snow depletion curves [26], statistics calculated from declines in SWE. Decline in snowcover extent based on changes in areal coverage of snowpack (Snow Covered Area) [26] is also used to measure snowcover loss in the spring. The area covered by snow is more relevant for albedo changes and the influence of solar radiation in warming, while SWE-based measures of decline in snowpack are the basis of estimates of water resource availability.

Climate change is projected to influence snowpack accumulation and ablation rates in the Rocky Mountains via increasing temperatures and a greater fraction of precipitation falling as rain during the traditional peak accumulation season [12, 27]. Studies have documented expected climate change linked reductions in western North American snowpack in recent decades [13, 28, 29] with up to 60% of snowpack decline between 1950 and 1999 attributable to human causes [30]. With correction for shifts in measurement methods and artifacts it is estimated that temperatures in the mountainous regions of western North America have already increased by more than 0.5°C [31]. Furthermore, a recent study showed that between 1950–2012 Western Canada experienced the most significant warming during the winter season and mean warming of 2°C [29]. Projections of future warming show a range of temperature increases up to 4-8°C by 2100 globally [31, 32] and 2-3°C plus in the Montana Crown of the Continent region during January-February-March (JFM) [33]. Increased occurrence of summer drought, an annual increase in precipitation of 3.3cm and a higher fraction of precipitation in winter as rain are projected for the Rockies of the border region of the US and Canada [33]. Thus, projected trends of warming temperatures and altered precipitation patterns will continue to impact water availability in snowmelt dependent watersheds [34, 35]. Precipitation, interacting with temperature and elevation, will be the ultimate determinant of winter snowpack accumulation in the COC region of the Rocky Mountains [4]. Moreover, studies have shown that extreme precipitation events have a large effect within the precipitation regime [36]. These extreme precipitation events tend to occur when temperatures are near freezing [37]. The balance between warmer temperatures causing more precipitation as rain and the occurrence of extreme winter precipitation events, however, is uncertain. Nonetheless, some projections anticipate that snowpack in the western United States will decline by as much as 60% in the next 30 years [34]. Managers will need tools to identify the level of expected watershed response to adapt to such dramatic changes in snowpack.

Despite the need for such tools, there is a dearth of studies that compare snowpack or water resources under coordinated management at local and region scales. Rood et al [38] developed comparisons of seasonal flow of watercourses within the Oldman River Basin. Buttle [39] examined basin storage in 5 adjacent basins in southeast Ontario. The hydrological consequences of bark beetle mortality in 7 Colorado River headwater catchments were studied by Biederman et al [40]. Snowpack and streamflow timing studies have been conducted at watershed (i.e., [41]), river basin [35, 42], regional [6, 13], national [26] and global [3] scales. Studies at intermediate bioregional scales spanning multiple river basins, however, are few (e.g., [11, 43]), yet managers need information at this scale to identify opportunities and challenges to climate adaptation and to resolve the allocation of resources.

### Snowpack data and analytical approaches

There are several common approaches to derive historic trends in snowpack. Analysis of long term empirical snowpack data from SNOTEL sites has been used to establish negative historical trends in SWE [13, 28]. However, matching the appropriate data needed for the scale of study is important and often difficult. For example, studies of individual watersheds can yield empirical estimates of SWE [25] taking into account local influences on accumulation and ablation, but once the scale expands to include multiple watersheds, extrapolation from dispersed point data (i.e., SNOTEL data], remotely sensed, or modeled data become preferred tools for estimation of snowpack characteristics. Comparison of three SNOTEL extrapolation datasets (Santa Clara, Daymet and CPC) found all were reliable data sources for lumped hydrological modeling at the basin scale [44]. The SNODAS dataset is derived from a combination of point observations, airborne and satellite observations and has been found to differ from watershed level observations yet perform satisfactorily at the basin-scale [45]. The ground weather station-based datasets PRISM [46] and Daymet [47, 48] have both been utilized in climate impact assessment, however, the Daymet dataset directly estimates SWE and extends into Canada and the US. Datasets derived at least in part from remote sensing, like SNODAS [49] or MODIS [50], are more recent in origin and are often limited by political boundaries and temporal extent, reducing their capacity to include the full range of climate variability including decadal regional and global processes.

The Daymet dataset grid coverage and resolution is appropriate for this analysis. Daymet uses interpolation to extrapolate point measurements of climate variables across the globe. In North America the source of the empirical data included in the version 2 model used here is from the SNOTEL network and from the Global Historical Climatological Network. Snow water equivalent is determined by precipitation accumulation when T_avg_ <_0°C. Data is available in 1 km^2^ pixels gridded across the analysis area. Daymet has been validated for moderate scale hydrological modeling previously [44]. Choices between watersheds in a regional context must be made to implement adaptation strategies, therefore, bioregional scale studies offer the best match between reliable data and management needs.

Either use of downscaled GCM projections of temperature and or historical data are options to investigate watershed level responses to climate change. Jones et al [8] integrated empirical stream temperature, land surface and climate data derived from Daymet with downscaled GCMs to estimate future stream temperature scenarios in the Crown of the Continent (COC) region of North America. Choices in modeling approach such as global climate models, emission scenarios, downscaling techniques, and hydrological models can significantly affect predicted hydrological outcomes [51]. Studies that have carefully used a suite of downscaled global climate models reduce uncertainty and provide hydrological information about trend over subcontinental scales (e.g., [52]). Historical data investigations of past watershed level responses to the variability of climate focus on April 1 SWE as the typical measure of snowpack accumulation used to estimate winter snowpack accumulation and summer water resource availability [13]. Historical snowpack data from SNOTEL sites has been used to document subcontinental trends in SWE over the last few decades [28, 34] and have been used to substantiate basin/watershed trends in streamflow [38]. Regional analyses using data on individual watershed SWE status are lacking, however.

This study utilizes multiple approaches to analysis of historical SWE to examine the historical effects of differing temperature and precipitation regimes on snowpack. The approach is to investigate relevant elevation, temperature and precipitation metrics, identify those linked most strongly with April 1 and June 1 SWE; determine the status of SWE in the warmest and driest years 1980–2013 in the COC; and the temperature and precipitation connections to higher levels of SWE retention under warmer and drier conditions and in later season conditions. Although snowfall continues in the high-elevation mountain regions after April 1, SWE generally peaks in March to early April [4] in the COC region of the Rocky Mountains. Knowles et al [12] found that over the period 1949–2004 in the western US the shift to a lower snowfall/total precipitation ratio was strongest in March. The snowmelt season in the COC continues well into June influencing streamflow and water availability in the early spring on into the late summer [35, 38]. In the Rocky Mountains near the US-Canada border the temporal centroid of streamflow occurs in May-June [6]. In this region, the months of April and May have higher average minimum temperatures and a greater fraction of precipitation as rain than February or March (see [53]), mimicking to some degree the expected shifts in peak snow accumulation season conditions in the future [27], notwithstanding the greater influence of shortwave energy in the later months on snowpack ablation. As a result, the examination of relative watershed-level persistence of SWE from April 1 to June 1 may inform adaptation to climate change at a scale useful to water management.

Comparative retention of SWE is relevant to adaptive landscape and water resource management choices. Resilience is the ability to maintain relationships within a system despite change in state variables [54] or the quality of being buffered from change [55]. Carey et al [43] defined catchment resilience as the ability to return to typical hydrological functioning following perturbation. Further, they defined the ability to store water for later discharge as catchment resistance. Therefore, research of the effects of elevation, temperature and precipitation on SWE is a first step toward understanding watershed response to climate change. This study aims to examine the relative SWE retention relationship of North American COC watersheds to elevation, temperature and precipitation variables and to later season (April1 to June 1) climatic conditions.

Here I use historical Daymet SWE estimates within COC watersheds to test response of COC snowpack to temperature and precipitation in two periods (January-February-March (JFM) and April-May) and to discover watersheds that best retain SWE to sustain later flows and under temperature/precipitation regimes like those expected with future climate change. This study also includes cross-border integration of information that transcends the data systems of a single country, evaluating a process that can be applied to integrated transboundary resource management throughout the world. The study’s objectives are to identify elevation, temperature and precipitation metrics linked to snowpack April 1 SWE, determine whether watersheds with higher relative SWE retention under warmer and dryer condition exhibit cooler temperature and/or wetter precipitation profiles than median SWE retention watersheds in the years of highest climate stress, examine relationships of later season (April-May) temperature and precipitation to higher SWE retention from April 1 to June 1, identify COC watersheds that possess high relative retention of SWE later in the peak flow season and following likely exposure to warming and precipitation under that warmer climate regime, and to validate the Daymet dataset through comparison with results using other datasets.

## Materials and methods

### Study site

**Fig 1:**
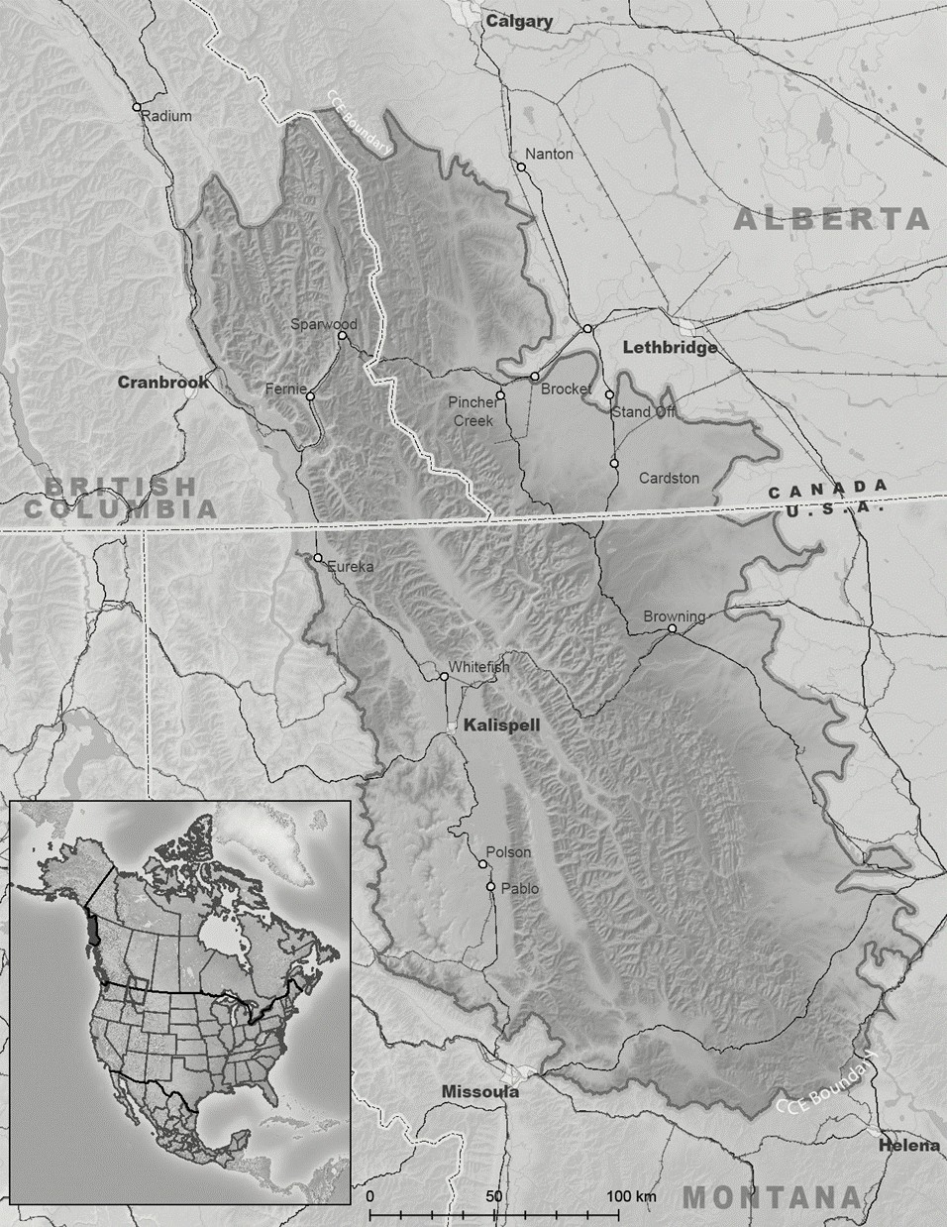
The Crown of the Continent study area. Map source is the Crown Managers Partnership data archive on the USGS Sciencebase data site: https://www.sciencebase.gov/catalog/item/51102e04e4b048b5cead853b.

The study was conducted within the boundaries of the North American COC Ecosystem (Fig 1) that spans the Canadian and United States Border on either side of the Continental Divide of the Rocky Mountains. The snowpack of this hydrological apex [8] of the continent feeds 3 major continental drainages: the Columbia, Missouri and Saskatchewan Rivers that flow to the Pacific, Atlantic and Arctic Oceans. The fate of snowpack within this region is therefore tied to water availability over a large portion of western North America. Elevation ranges from 740m to 3338m and precipitation from 40 cm/year to 350 cm/year. The western side of the region is bounded by the Rocky Mountain Trench and climate is governed by Pacific Northwest Maritime systems [56]. The eastern side falls precipitously from the Continental Divide to high plains and climate is controlled by continental systems from the north and south [11]. Topography is generally complex belt series mountainous terrain shaped by glacial processes that began retreat some 15000 years ago and continue today at the highest elevations [32]. High alpine tundra transitions to dense conifer dominated forests that give way to grasslands on the eastern slopes and in the western valleys. The COC has significant natural, undeveloped watersheds in the extensive protected area network within its boundaries. Orographic precipitation patterns produce a pronounced eastern rain shadow effect. The region’s long term climate is influenced by the Pacific Decadal Oscillation and annual variations in the El Niño Southern Oscillation [29, 57]. Northwestern North America, including the COC, has also been identified as a region of significant warming over the last century [58].

### Dataset

The Daymet 2.0 dataset [47, 48] was selected for its longevity, availability across international borders and scale. Daymet datasets for 1980–2013 were downloaded from the Oak Ridge National Laboratory Distributed Active Archive Center [49] and processed for use in ArcGIS. The Daymet dataset corresponds with a documented period of decline in snowpack [28] and calculates SWE (kg/m^2^) daily on a 1 km^2^ grid [49, 58]. Daymet depends on daily minimum temperature (Tmin, °C), maximum temperature (T_max_, °C) and precipitation (mm) measures from SNOTEL stations to interpolate across larger regions taking into account elevation in complex topography [59]. The snowpack model accumulates snow when precipitation occurs and (T_max_ + T_min_)/2 ≤ 0.0°C. Snowmelt occurs at a calibrated rate when T_min_> T_crit_ where T_crit_ (°C)is a calibrated threshold of snowmelt. The algorithm and resulting estimates were validated in an alpine region of Austria [59] and has also been validated for moderate scale analyses of hydrology. April 1 Daymet estimates were checked for correspondence with empirical SWE measures from the 14 SNOTEL precipitation gauges and the SNODAS data for the years 2010–2014 in the Montana, USA portion of the COC using Kendall tau [60] and least squares regression to assess whether the Daymet estimates were valid indicators of comparative SWE.

Watershed boundaries were mapped at the 10^th^ code Hydrologic Unit Code (HUC) level across the COC. A challenge for watershed analyses that transcend political boundaries is dataset compatibility, especially scales of available GIS data. For this reason I combined available digital watershed delineations. The USGS National Watershed Boundary Dataset [61] provides one watershed delineation dataset that spans the Montana and southeastern British Columbia portions of the COC region. Most of the Alberta portion of the COC had been mapped by the Oldman Watershed Council at a finer resolution (Fiera Consulting 2016). These watersheds were combined to approximate the HUC10 scale using topographic maps available from the GeoGratis site of Natural Resources Canada [62] and the COC Streams dataset developed by the Crown Manager’s Partnership [61]. Alberta watersheds outside the Oldman River basin were mapped from the National Hydro Network 1.0-CL1_NC1 dataset [63].

### Analytical approach

Daymet estimates of overall COC temperature and precipitation values were derived using zonal stats in spatial analyst in ArcGIS. Mean minimum and maximum temperature (T_min_ and T_max_) for January, February and March were determined separately and also combined to estimate winter values for those metrics. Monthly T_max_ and T_min_ showing an association with April 1 SWE were averaged for each period to derive the metric T_avg_. Mean total COC and watershed mean daily precipitation (mm/day) for JFM were summed to create a winter precipitation value for comparison. Mean elevation (elevation) for each watershed was gained through ArcGIS Spatial Analyst using a digital elevation model [64]. Elevation, temperature and precipitation measures were tested by least squares regression and the Kendall non-parametric test [60] for correlation with COC annual April 1 mean SWE per ha from 1980–2013. Those measures proving influential (adjusted r^2^≥0.10, p<0.05) were then used to divide the SWE data into the 11 warmer or drier years or the 23 cooler or wetter years respectively.

Values for inter-watershed comparison were derived by watershed. Snow water equivalent (kg/m^2^), temperature (T_min_ and T_max_ °C), precipitation (mm/day) values for April 1 and June 1 of each year 1980–2013 were derived for each watershed using ArcGIS Spatial Analyst. The SWE values for all grids within the polygon were summed for April 1 and June 1 separately for each year and divided by the area of the polygon in ha to derive an area independent measure (kg/m^2^/ha) for comparison. Watersheds with ≥50% of the June 1 SWE values of 0 were eliminated from the analysis. One hundred twenty one watersheds were analyzed. The mean SWE for April 1 and June 1 for each watershed polygon was then calculated. Retention of snowpack was estimated for the spring season (spring retention), the 11 warmest years 1980–2013 (warm retention) and the 11 driest years 1980-2013(dry retention) (Table 1).

**Table 1:** Snowpack retention metrics.

Metric	Estimation Method
**Warm retention**	[(mean warm year April 1 SWE/ha)/(mean cooler year April 1 SWE/ha)]*100
**Dry retention**	[(mean dry year April 1 SWE/ha)/(mean wetter year April 1 SWE/ha)]*100
**Spring retention**	[(June 1 SWE/ha)/ (April 1 SWE/ha)]*100

The 11 warmest or driest years 1980–2013 in the COC were determined by COC wide T_max_ in February and COC wide lowest mean precipitation in mm/day JFM respectively. To measure retention of SWE in warm or dry years compared to cooler or wetter years or retention of April 1 SWE to June 1 a ratio of April 1 SWE in warm/dry to April 1 SWE cooler/wetter is derived and converted to a percentage for each watershed. Spring retention is the ratio of June 1 SWE to April 1 SWE across the entire period for each watershed.

Quartiles are used in snowpack monitoring data analysis to elucidate current snowpack SWE values in water resource versus historic SWE management (see [65]) and in other hydrologic/snowpack assessments [23, 66]. Differences between T_min_, T_max_, T_avg_ and precipitation values for the top 30 watersheds (top quartile -1) versus the middle 30 watersheds were tested using the non-parametric Wilcoxon rank sum test [67]. Watershed SWE values were ranked by warm and dry retention in the 11 warmest and driest winters respectively in the analysis period compared to the cooler/wetter winters to identify watersheds with the best retention of snowpack across the warmest and driest conditions of the period 1980–2013 and by spring retention across warm, dry and all years to gauge summer water availability and as a proxy for future warmer conditions.

Three comparisons of the resulting data were made with other datasets to validate the results. Trend in overall April 1 SWE was examined using least squares regression of 5 year running means of overall COC SWE. Daymet data were also compared with data analyzed for the same watersheds from other sources. SWE datasets for April 1 2010–2014 were obtained from the Snow Data Assimilation System (SNODAS). Snow water equivalent is estimated in meters using remote sensing data independent of the SNOTEL system. To control for issues of scale and data spatial scope, comparison was made using April 1 SWE for the USGS watersheds only. Correspondence of SWE values between Daymet and SNODAS datasets was tested using least squares regression and Kendall correlation. Finally, the Daymet results here were compared with average SNOTEL values of April 1 SWE within 1980–2013 for sites in the United States portion of the COC using least squares regression and Kendall correlation. Analysis was limited geographically to ensure uniformity of SNOTEL equipment and protocol by using a single jurisdiction and because the largest number of watersheds with SNOTEL data occurred in that jurisdiction. Only SNOTEL sites with 20 years or more of SWE data within the period were used in the analysis. Comparison was made of the Daymet grid value corresponding to the SNOTEL site location.

## Results and discussion

The COC exhibited the substantial SWE measures expected of the hydrological apex of 3 major river systems. In 1980–2013 April 1 SWE ranged from 0.11 kg/m^2^/ha to 4.44 (mean = 1.66, median = 1.55, SD = 1.02). Spring retention varied widely across the watersheds. The highest spring retention reported was 97.23% (Elk River Headwaters) and the lowest was 7.79% (mean = 51.85%, median = 57.5%, SD = 23.20) in the 121 watersheds analyzed. Percent retention rank has 31 watersheds in the ≥ 75^th^ percentile with a low value of 67.36% mean loss of SWE April 1 to June 1. Warm retention ranged from 27.8% to 138% (mean = 80.53%, median = 82.08%, SD = 15.62%) indicating that some watersheds had greater snowpack in the warm years. Dry retention showed more substantial effects, ranging from 25.18% to 74.53% (mean = 58.19%, median = 59.24%, SD = 8.71%). Daymet results for April 1 SWE corresponded with other datasets and trends established by other studies. Correlation of the Daymet estimated April 1 SWE with empirical US SNOTEL observations within the same period and region of the COC was positive and highly significant (r^2^ = 0.51, p<0.01). Likewise, Daymet April 1 SWE was positively correlated with SNODAS April 1 SWE (r^2^ = 0.62, p<0.001).

Between temperature and precipitation influence on 1 April SWE at the full COC scale, precipitation was the more significant factor. None of the temperature metric (T_min_, T_max_, or T_avg_) means showed a dual significant correlation with April 1 SWE at that scale, although February T_min_ and T_max_ were significantly correlated in linear regression alone (Table 2). Kapnick et al [68] found February to be the weakest month for temperature correlations and monthly SWE values. In contrast, the COC has the strongest relationship between February temperatures and April 1 SWE. Mean precipitation in JFM did positively influence April 1 SWE (Table 2). Precipitation in January and February also showed a positive association with 1 April SWE (Table 2). The lack of association between temperature and April 1 SWE is striking given the several studies that have found temperature was a major influence on SWE [2, 13, 69]. Hamlet et al [70] found that continental areas of western North America, like the COC, demonstrated stronger linkage with winter precipitation trends in determining April 1 SWE, however. They attributed this pattern to colder winter temperatures resulting in low temperature sensitivity. Fassnacht et al [71] found that SWE tracked precipitation values through the winter in the Rocky Mountain National Park area, a scale akin to this study. These results might also differ because other studies rely on the use of SNOTEL stations generally found in a narrow elevation band compared to use of an interpolated dataset here to expand those empirical measurements across a wider band of elevations. Sospedra-Alfonso et al [72] noted strong positive elevation influence on snowpack and the predominance of precipitation driving snowpack at higher elevations (>1560m) in the Idaho-Montana Columbia River Basin. Likewise, Mote [73] noted that temperature predominated at lower elevations and precipitation at higher elevations. Median mean watershed elevation in the COC is 1572 m and median elevation of the highest ranked retention watersheds exceeded the 1560 m elevation threshold for precipitation dominance in SWE accumulation (Table 3). The importance of precipitation has emerged in some western North American mountain region studies at larger scales [72, 74, 75] and these findings are consistent with those studies.

**Table 2:** Regression and Kendall correlation matrix values for Crown of the Continent temperature and precipitation metrics.

		*April 1 SWE*		*Spring Retention*	
		*r*^2^(T)	*P*	*r*^2^(T)	*P*
*Precipitation*	JFM	0.377 (0.483)	<0.001(<0.001)**	0.186(0.276)	0.009(0.022)**
	Jan	0.387 (0.458)	<0.001(<0.001)**	-	-
	Feb	0.241 (0.341)	0.002 (0.004)**	-	-
	Mar	-0.022(0.162)	0.601 (0.184)	-	-
	April	-	-	0.050(0.175)	0.106(0.146)
	May	-	-	0.241(0.185)	0.170(0.123)
*T*_*min*_	Jan	-0.032(0.11)	0.929(0.36)	-	-
	Feb	0.140(-0.19)	0.017(0.11)*	-	-
	Mar	-0.006(-0.13)	0.371(0.302)	-	-
	Apr	-	-	-0.024(0.02)	0.645(0.88)
	May	-	-	0.134(-0.344)	0.018(0.004)**
*T*_*max*_	Jan	-0.021(-0.005)	0.563(0.977)	-	-
	Feb	0.135(-0.159)	0.02(0.194)*	-	-
	Mar	-0.010(-0.09)	0.413(0.462)	-	-
	Apr	-	-	-0.029(-0.037)	0.790(0.76)
	May	-	-	-0.217(-0.173)	0.278(0.156)
*T*_*avg*_	Feb	0.140(-0.212)	0.017(0.080)*	-	-
	JFM	0.068(-0.119)	0.074(0.33)	-	-
	Apr	-	-	0.002(-0.013)	0.310(0.93)
	May	-	-	0.058(-0.19)	0.091(0.41)

Correlation with April 1 SWE and/or spring retention are shown (N = 121). The regression coefficient *r*^2^ and Kendall tau (T) are shown outside and inside parentheses respectively. Kendall correlation p values are in parentheses. Significance for both linear regression and Kendall correlation is shown by double asterisks and single asterisks denote correlations significant only for linear regression.

**Table 3:** Temperature, precipitation and elevation influences on warm or dry retention.

Metric	Month	Type	Warm or Dry Retention			
			Median		*p*	W
			*Top*	*Mid*		
T_min_(°C)	Feb	Warm	-3.18	-3.09	0.631	483
T_max_(°C)	Feb	Warm	2.408	4.469	<0.001	666.5
T_avg_(°C)	Feb	Warm	-0.489	0.866	0.003	158
**Total Precipitation (mm/day)**	JFM	Dry	55.87	45.96	0.002	644
**Elevation (m)**	-	Warm	1768.9	1543.5	0.007	632
		Dry	1774.4	1612.1	<0.001	677

Results shown for the top 30 watersheds and the middle 30 watersheds ranked by warm and dry retention. The Wilcoxon rank-sum W statistic for each comparison is reported.

The results here suggest elevation is playing a predominant role in shaping temperature and precipitation influence in the COC as well. Elevation had the strongest association with both April 1 SWE and spring retention at the watershed scale and that pattern continued in warm and dry years, consistent with the many studies that demonstrate such effects [72, 76] (Table 4). Mean watershed elevation had a strong effect on June 1 SWE (r^2^ = 0.412, p<0.001;) as well. Elevation will remain constant despite climate change and the strong influence it has on temperature, precipitation and, consequently, snowpack accumulation/ablation will likely continue. Whether temperature effects of climate change will ultimately progress to the highest elevations is uncertain, but the high relative elevation of the COC promises to buffer those effects to some extent.

**Table 4:** Regression and Kendall correlation matrix values for elevation at the watershed scale.

		*April 1 SWE*		*Spring Retention*	
		*r*^2^(T)	*P*	*r*^2^(T)	*P*
*Elevation*	-	0.499(0.538)	<0.001(<0.001)	0.369(0.285)	<0.001(<0.001)
	Warm	0.459(0.464)	<0.001(<0.001)	-	-
	Dry	0.422(0.463)	<0.001(<0.001)	-	-

Correlation with April 1 SWE and/or spring retention are shown (N = 121). The regression coefficient *r*^2^ and Kendall tau (T) are shown outside and inside parentheses respectively.

Unlike elevation, latitude was not prominent in influencing snowpack at the watershed scale in the COC. Latitude was indicative of Daymet rankings at the watershed scale, but did not explain the majority of variation in the data. Latitude, although significantly correlated with April 1 SWE (r^2^ = 0.04, T = 0.156, p<0.05,) spring retention (r^2^ = 0.072, T = 0.214, p<0.01), and June 1 SWE (r^2^ = 0.028, T = 0.137, p<0.05), has a much weaker effect on snowpack across the COC.

Elevation, temperature and precipitation were markedly different in the highest warm or dry retention watersheds, however. Median mean elevation was more than 200 meters higher in the highest warm retention watersheds than mid-ranked warm retention watersheds (Table 4). In dry years elevation did not differ as much (162.3 m), but that difference was more strongly significant (Table 4). Consistently, the top warm and dry retention watersheds showed higher JFM precipitation and lower February T_avg_ than median watersheds (Table 3). Thus, as expected, elevation, temperature and precipitation play a major role in determining April 1 SWE and warm and dry retention at the watershed scale. Overall trends in the literature include declining precipitation with increased temperature at regional scales (e.g., [77]). Therefore, it is worth noting that temperature and precipitation are significant factors driving SWE and its retention at the watershed scale under the warmer conditions expected under climate change.

The mean March and May T_min_ and T_max_ values show strong warming historically 1980–2013. March (-8.49°C)-May (-1.69) T_min_ rises by 6.8°C and T_max_ (-1.69 and 12.04 respectively) by 13°C. Average temperature in those two months shows an 8.44°C warming. These values exceed the temperature rise maximum seen in many climate models under a business as usual scenario [31, 78]. Therefore, spring retention represents an extreme of estimated future climate temperature change. The retention of SWE from April 1 to June 1 does, however, represent water held in snowpack at the peak of runoff and signals the potential for summer streamflows [35, 38, 77].

The later season snowpack analysis found that temperature was a stronger influence on spring retention at the COC scale. A study of western North America found that correlations between temperature and later periods in the snow season (post April 1) strengthen [68]. The trend of increasing negative correlations between temperature and SWE the later in the snow season [68] is reflected in the correlation between May T_min_ and spring retention of snowpack in the COC (T = -0.344, p = 0.0039). Fassnacht et al [75] found positive correlations between April and May temperatures and bi-monthly snowmelt rates, contrary to the April temperature disconnect from spring retention and consistent with the May T_min_ correlation with spring retention in the COC. April temperatures may be too remote from June 1 conditions or overwhelmed by intervening events like rain on snow precipitation to significantly influence that metric. May mean precipitation had a slight positive effect on Jun1 SWE (r^2^ = 0.092, p = 0.045; T = 0.2676, p = 0.0262). It has been postulated that higher elevation sites remain cold enough to allow late season precipitation as snow to counter increased temperatures and that late season storms can therefore lead to higher accumulation [68]. In the COC late season precipitation can slow the loss of SWE. The remaining temperature metrics did not show significant correlations to Jun1 SWE or retention (Table 2), perhaps due to the increased influence of short wave radiation, longer day length, aspect and increased temperature overall.

Late spring watershed scale responses to warm and dry years followed the pattern leading to differences in April 1 SWE and warm/dry retention. Both greater Apr-May precipitation and lower T_avg_ were associated with spring retention in dry and warm years (Table 5). Late spring precipitation was positively associated with higher retention in warm years and lower T_avg_ found in the top 30 spring retention watersheds during dry years, suggesting potential combined effect of precipitation and temperature to abate snowpack loss in unfavorable climate years. The latitudinal variation encompassed by the COC could be contributing to this pattern as the snowmelt season shifts later from the southern to northern edge of the region (see [25]).

**Table 5:** Temperature and precipitation influences on spring retention.

Metric	Month	Warm Yr Spring Retention				Dry Yr Spring Retention			
		*Top*	*Mid*	*p*	*W*	*Top*	*Mid*	*p*	*W*
T_min_(°C)	May	-3.37	-1.72	<0.001	107	-2.45	-1.22	<0.001	198.5
T_max_(°C)	May	8.75	11.98	<0.001	147.5	9.5	12.85	<0.001	141
T_avg_(°C)	May	3.15	4.58	<0.001	187	3.97	5.32	<0.001	226
**Total Precipitation (mm/day)**	Apr	98.86	70.45	<0.001	745	99.5	70.27	<0.001	709
	May	93.65	78.21	<0.001	725.5	93.95	78.21	<0.001	710.5
	Apr-May	86.29	69.13	<0.001	702.5	84.25	67.61	<0.001	712

Results shown for the top 30 watersheds and the middle 30 watersheds ranked by spring retention of SWE overall. Wilcoxon rank-sum W statistic for each comparison is reported.

Factors influencing snowpack in the COC operate at more than one scale. Temperature has been established as a leading factor linked with snowpack condition in many sub-continental scale studies [13]. Here, temperature operates to shape snowpack condition at the watershed rather than regional scale when conditions are least favorable for winter snowpack accumulation and spring retention. Precipitation emerges as a substantial influence at both the regional and watershed scale in both winter and spring. Kapnick and Hall [68] found that interannual temperature variation in February had little influence on April 1 SWE, but found March-May temperature affects spring SWE accumulation and melt events in a regional average temperature analysis. The COC exhibits the same lack of significant regional correlation for JFM winter snow season temperature.

**Fig 2:**
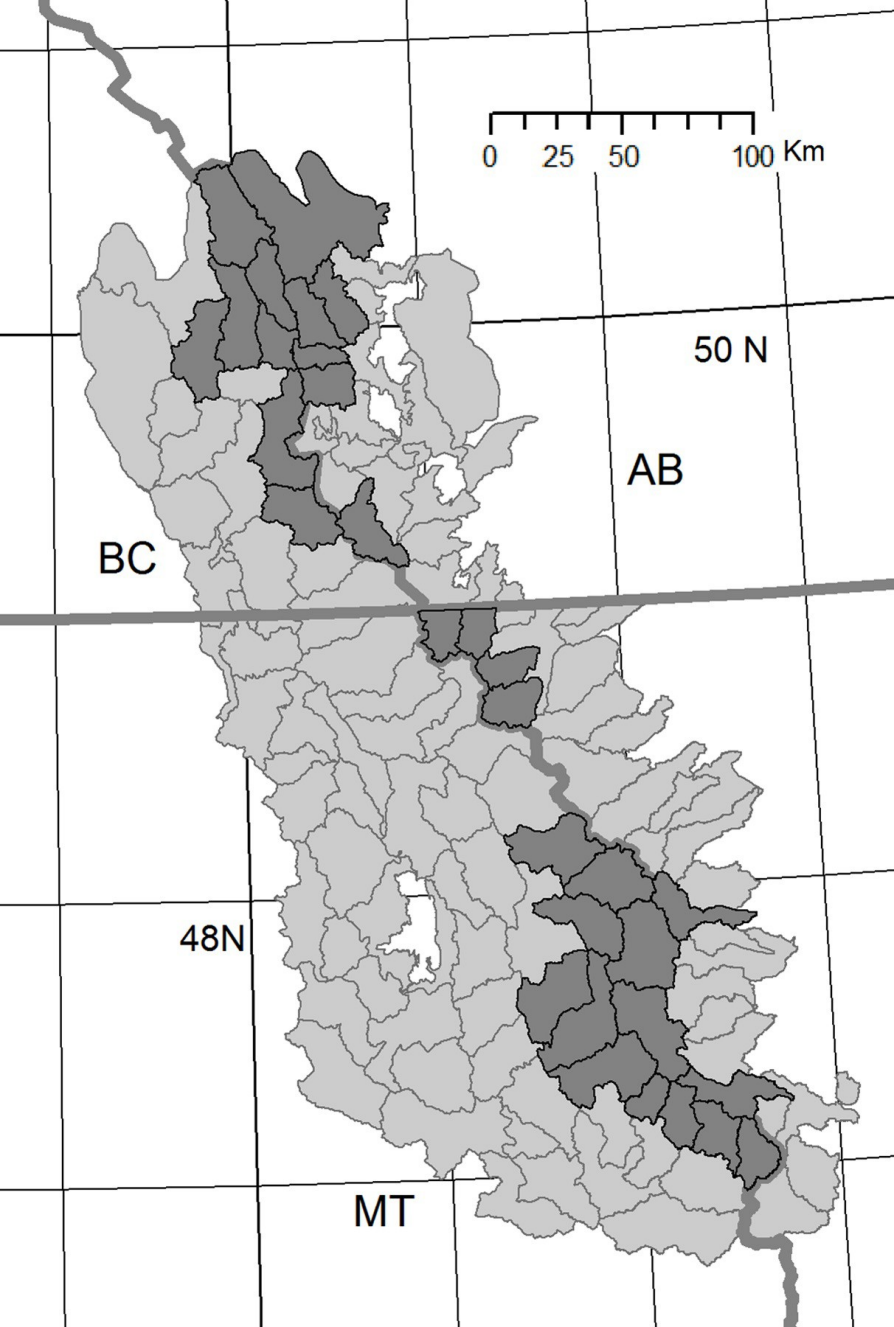
Highest mean elevation watersheds in the Crown of the Continent. Dark shaded watersheds indicate >75^th^ percentile ranked watersheds based on mean elevation determined from the Crown Managers Partnership digital elevation model. Contains information licensed under the Open Government Licence-Canada.

**Fig 3:**
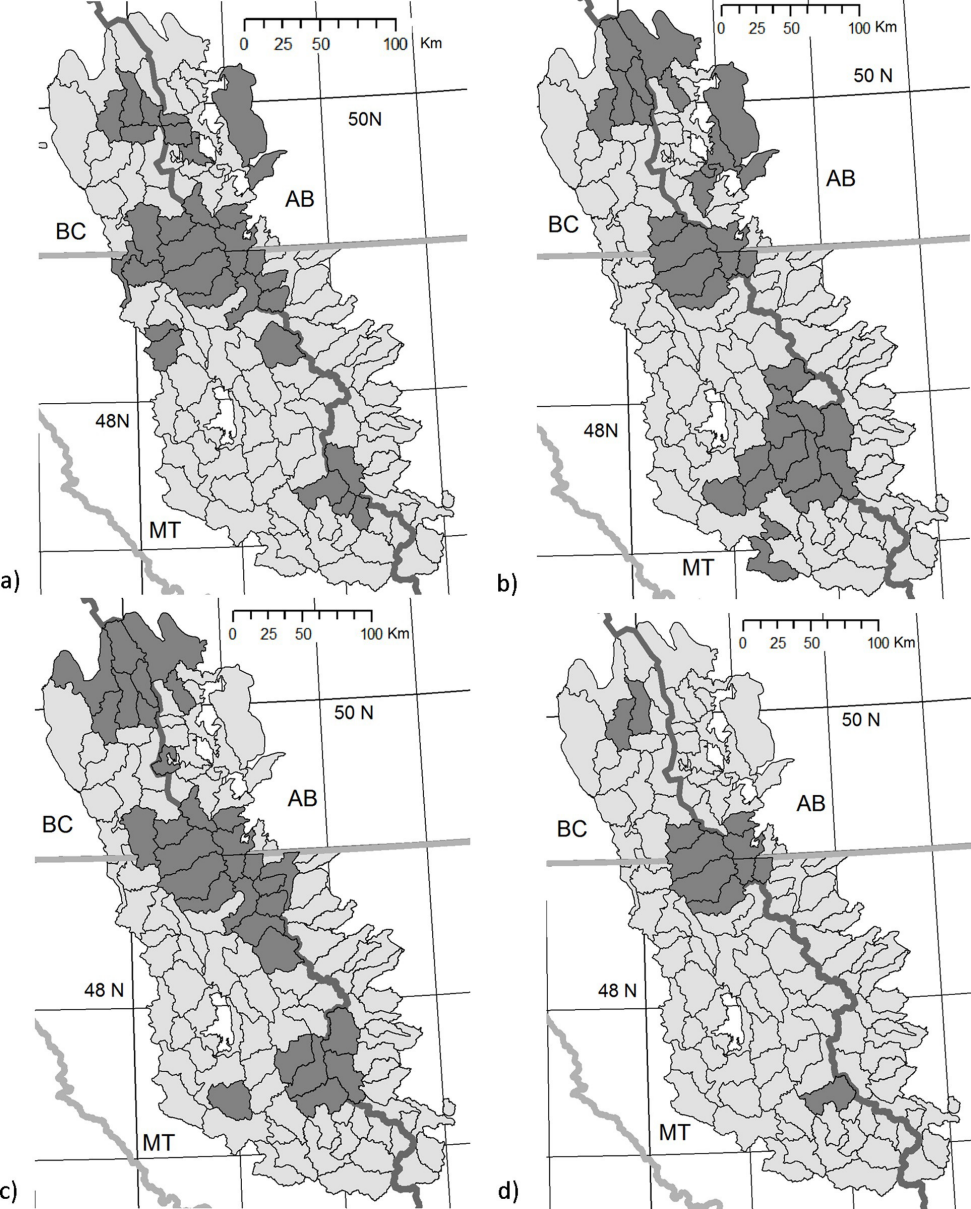
Crown of the Continent high retention watersheds as measured by warm, dry and spring retention of SWE. Dark shaded watersheds indicate >75^th^ percentile ranking for: (a) warm retention, (b) dry retention and (c) spring retention. Watersheds ranking high for all 3 measures of retention are shown in (d). Contains information licensed under the Open Government Licence-Canada.

Assessment of watershed resistance to snowpack loss under higher temperature or drier conditions can assist identification of watersheds most likely to resist anticipated climate change effects. Climate models project higher winter and spring temperatures as well as less precipitation as snow [33, 79]. Higher winter/spring temperatures are predicted with high confidence, but the changes in precipitation are less so (see [80]). For the COC region predictions are for greater total precipitation January-May, but it is expected to be more often as rain. Nonetheless, watersheds that possess greater snowpack accumulation and resistance to early snowpack loss will be important as refuges for coldwater species and as sources of summer streamflows in these snowmelt dependent systems. By combining multiple measures of relative watershed snowpack performance in years with higher winter/spring temperatures, drier precipitation regimes, and spring retention of snowpack, watershed climate adaptation values can be assigned to select high-resistance watersheds. As expected, the highest mean elevation COC watersheds (Fig 2) are grouped closer to the Continental Divide in general. Fig 3 illustrates the watersheds with higher performance in warm years, dry years, spring snowpack retention and under all those metrics combined. Despite the strong influence of elevation, comparison of the top 30 watersheds for warm, dry or spring retention shows that 16, 15 and 14 of the top watersheds for the climate related metrics fall outside of the top mean elevation watersheds. Thus, elevation alone cannot serve as a surrogate for historical performance of snowpack retention under less favorable conditions. Of the top quartile of watersheds in warm, dry or spring retention, 11 watersheds are high SWE retention watersheds in all those unfavorable snowpack retention conditions. Another 18 watersheds are top quartile watersheds for two of those measures. Together, these watersheds offer high relative climate change adaptation potential under relevant conditions based on their historical record.

Use of the Daymet dataset allows greater resolution of climate change retention at the watershed scale. Ten years later, the assertion of Carey et al [43] that few comparative catchment studies exist appears to remain true. Other assessments of the past and projected trends in snowpack have been done in western North America, but most have operated at the basin scale or higher [81–83]. One prior study focused on the COC and used SNOTEL and snowcourse data from 33 locations [11]. Limited by the comparability of data, only one location was in Canada and the assessment established trends for the southern portion of the COC spanning many watersheds. A Montana assessment sampled a subset of river basins for the entire state as representative of climate regions outside the COC [82]. A British Columbia assessment [83] used the ecoprovince scale documenting a 5% per decade decline in April 1 SWE for the Southern Interior Mountains province. These assessments and studies provide valuable information to inform managers but do not allow managers to adapt at the watershed scale. Thus, this study provides a method for comparing watershed SWE retention heretofore absent from the literature.

Fewer studies have used interpolated datasets such as Daymet due to their limitations. Oyler et al [31] identified temperature biases in SNOTEL records that are the basis for interpolation in Daymet resulting in overestimated temperature increases for the past several decades. Yet, few datasets exist with the fine scale estimation of SWE values spanning wide geographic areas. The recent development of a more robust western US SWE dataset by the University of Arizona [84] is promising but it does not transcend the US Canada border and would therefore limit analysis in a coordinated management landscape. Daymet is used here to explore its utility as a relative watershed SWE and contributing factors dataset. It is not suitable for absolute parameter estimation. The aim of the study here is to provide information relevant to management decision-making that must proceed even if data is incomplete or uncertain. Since the advent of this study the Daymet dataset has been updated, offering an opportunity to more reliably estimate snowpack parameters.

Precipitation estimates used in this study are mean precipitation per day rather than precipitation as snow or the snow/precipitation fraction. Estimation of the snow/precipitation fraction would require daily estimation at a minimum, substantially increasing analysis time and duplicating steps already encompassed in Daymet SWE estimates. Many studies rely on total precipitation. Nonetheless, relationships of precipitation as snowfall to SWE would likely be stronger.

Spring snowpack ablation is influenced substantially by shortwave radiation [20, 72] that is not accounted for here. Shortwave radiation is difficult to establish over the scale of this study and researchers have often resorted to temperature-based models as a result [68]. The strength of association between elevation, temperature and precipitation metrics suggest that other factors are acting to influence snowpack persistence in the COC and investigation of the influence of shortwave and longwave radiation on ablation might be fruitful.

Choice of factors to include in watershed climate change resistance is to some degree subjective and the metrics chosen here could be altered to incorporate stronger datasets available for the area of interest and dependent on the aims of the adaptation process. In this study April 1 SWE retention was used as the metric to emphasize watersheds deemed most likely to resist warm or dry year conditions that might be expected to increase. While April 1 SWE is more traditionally used as the measure of snowpack integrity, a relative measure is more justified given the nature of the Daymet dataset rather than use of an absolute estimate like April 1 SWE. Total precipitation is likely to shift to greater fractions as rain, but may also be greater in total, making use of dry years less certain. Increased numbers of winter rain on snow events may accelerate snowpack ablation in unpredictable ways. Moreover, the greater uncertainty of precipitation projections in general add uncertainty.

## Conclusion

The results of this study, in a key hydrological region of North America, add to the limited literature contrasting individual watershed performance. While elevation has long been recognized as a major factor mediating snowpack accumulation and ablation, these results tie elevation strongly to watershed level differences in SWE and suggests that more studies linking elevation with temperature and precipitation effects are needed at the watershed scale. This study also joins a body of research indicating the importance of precipitation in driving SWE accumulation and later season SWE retention in mountainous landscapes (see [13, 70, 73, 75]). Daymet derived estimates of SWE show strong linkages to elevation in all conditions at the watershed scale. Drier and warmer years possibly enhance the effects of elevation in retaining snowpack in the COC under unfavorable conditions. Precipitation likely plays a stronger role than temperature in determining snowpack retention in the COC historically, perhaps because of COC elevation straddling the Continental Divide.

By examining retention of snowpack under warmer and drier conditions that may result from climate change and combining spring retention of snowpack, watersheds with higher historical SWE retention most robust to expected future changes can be distinguished. Spring retention of snowpack that will continue to yield substantial water resources into the warmer seasons can also be identified, aiding adaptation. To date, managers have had limited tools, often requiring extensive computation efforts to derive data indicative of the relative performance of mountainous watersheds within a region of hydrological importance. The approach used here offers a GIS option to gain initial information useful to estimating relative value of watersheds for climate change adaptation measures. While Daymet estimates may not tightly conform to empirical observations, they can still be reliably used to distinguish relative watershed performance, especially in headwater basins that transcend national borders, a comparison currently unavailable through the use of more limited empirical observations.

## Supporting information

S1 File (XLSX)

S2 File (XLSX)
